# Changes in the oral status and periodontal pathogens in a Sardinian rural community from pre-industrial to modern time

**DOI:** 10.1038/s41598-022-20193-9

**Published:** 2022-09-23

**Authors:** Eleonora Casula, Maria Paola Contu, Cristina Demontis, Ferdinando Coghe, Giorgio Carlo Steri, Alessandra Scano, Maria Laura Ferrando, Germano Orrù

**Affiliations:** 1 Section of Pharmaceutical Sciences, Department of Life and Environmental Sciences, University of Cagliari, Cagliari, Italy; 2 Molecular Biology Service (AOU-Cagliari), Department of Surgical Sciences, University of Cagliari, Cagliari, Italy; 3 Laboratorio Analisi Clinico-Microbiologiche, Azienda Ospedaliera Universitaria (AOU), Monserrato, Cagliari, Italy; 4 Azienda Per La Tutela Della Salute (ATS), Aree Socio-Sanitarie Locali (ASSL) of Cagliari, 09131 Cagliari, Italy; 5 Present Address: Emerging Bacterial Pathogen Unit, IRCCS San Raffaele Scientific Insitute, Milan, Italy

**Keywords:** Biological techniques, Microbiology, Molecular biology, Diseases

## Abstract

The oral microbial profile in humans has evolved in response to lifestyle changes over the course of different eras. Here, we investigated tooth lesions and the microbial profile of periodontal bacteria (PB) in dental *calculus* of a Sardinian pre-industrial rural community. In total, 51 teeth belonging to 12 historical individuals buried in an ossuary in the early 1800s and 26 modern teeth extracted from 26 individuals from the same geographical area were compared to determine the oral health status, bacterial load and amount of most relevant PB. Total caries and bacterial genomes count appeared to be sex-related in historical samples. Historical females presented a higher incidence of caries, PB pathogens and a higher bacterial load than historical males. Furthermore, we compared the PB profile of the historical individuals with the modern ones, revealing a notable increase in modern individuals of PB belonging to “*Red complex bacteria”* often associated with periodontitis and other chronic diseases of modern life. Our findings could be explained through an analysis of environmental factors such as socioeconomic, hygienic and healthy conditions that can have a great impact on oral health and bacterial composition among individuals of the same and different eras.

## Introduction

The analysis of dental wear, chemical composition and carious lesions of historical teeth can provide information about the lifestyle of ancient populations^1,2^. Archaeological microbiology is expanding through the investigation of ancient DNA analysis of oral bacteria recruited within mineralized concretions of teeth^3^. The subgingival and supragingival plaque of the teeth is rich in calcium phosphates and silicates, which can calcify in situ during the life of the host^4^, forming layered fossilized concretions known as dental *calculus*^5^. These concretions incorporate human and microbial DNA, dietary, and other environmental materials, preserving them from taphonomic and environmental alterations and keeping them intact for millennia^6^. Morphological examinations and genomic analysis of dental *calculus* of ancient humans can reveal how the microbial composition, diet and oral health in historical populations differs from that of modern humans^7–12^.

The health status of the host and the oral microbial composition are mutually related^13^. In recent years, medical/dental studies have been directed on some oral bacterial species potentially pathogenic known as periodontal bacteria (PB). PB are responsible for severe periodontal diseases and appear to play a possible role in the onset of several chronic critical illnesses, such as cardiovascular diseases, rheumatoid arthritis and intestinal cancer^14–16^.

The most common PB pathogens are grouped into several bacterial complexes based on their pathogenicity: (1) Socransky’s “*Red complex bacteria*”, which include the most highly pathogenic PB, such as *Porphyromonas gingivalis, Tannerella forsythia,* and *Treponema denticola*; (2) *“Orange complex bacteria,* including *Fusobacterium nucleatum* and *Prevotella intermedia,* which form a link between the early colonizers and highly pathogenic PB; and (3) *Aa-complex* comprising *Aggregatibacter actinomycetemcomitans,* which, together with *Red complex bacteria,* can drive the destruction of the soft tissue and bone^17^.

These bacterial species are normally considered opportunistic PB and are found in the biofilm of the tongue or supragingival plaque^18^. However, under certain changes in environmental conditions, they can grow as biofilms in the subgingival pockets, causing severe inflammation of soft gingival tissue and bone^19^. Some researchers have suggested that the growth of PB in the oral cavity is also influenced by external factors, including oral hygiene and dietary and smoking habits^5,20,21^. The current possibility of quantifying these bacteria in dental *calculus* through advanced real-time polymerase chain reaction (RT-PCR)^2^ represents a sensitive tool for the evaluation of oral health of an individuals^22^. Only a few studies have investigated whether the communities of pathogenic bacteria causing periodontitis are different between historical and modern populations^23^. This study aims to verify the difference in oral status within and between dental samples derived from individuals living in the same geographical area 200 years apart from each other. This comparison could be particularly interesting due to the peculiarity of the Sardinian population, which has been genetically stable for centuries but has undergone a significant lifestyle change over the course of the last 200 years^24^.

## Methods

### Villaputzu site and sampling procedure for human remains

The archaeological samples were recovered from a closed charnel house that, according to historical documentation, was coeval with human remains. It is found in Villaputzu, a small town located in the Sardinian *Sarrabus* region (Italy), a territory historically highly isolated.

**Figure 1:**
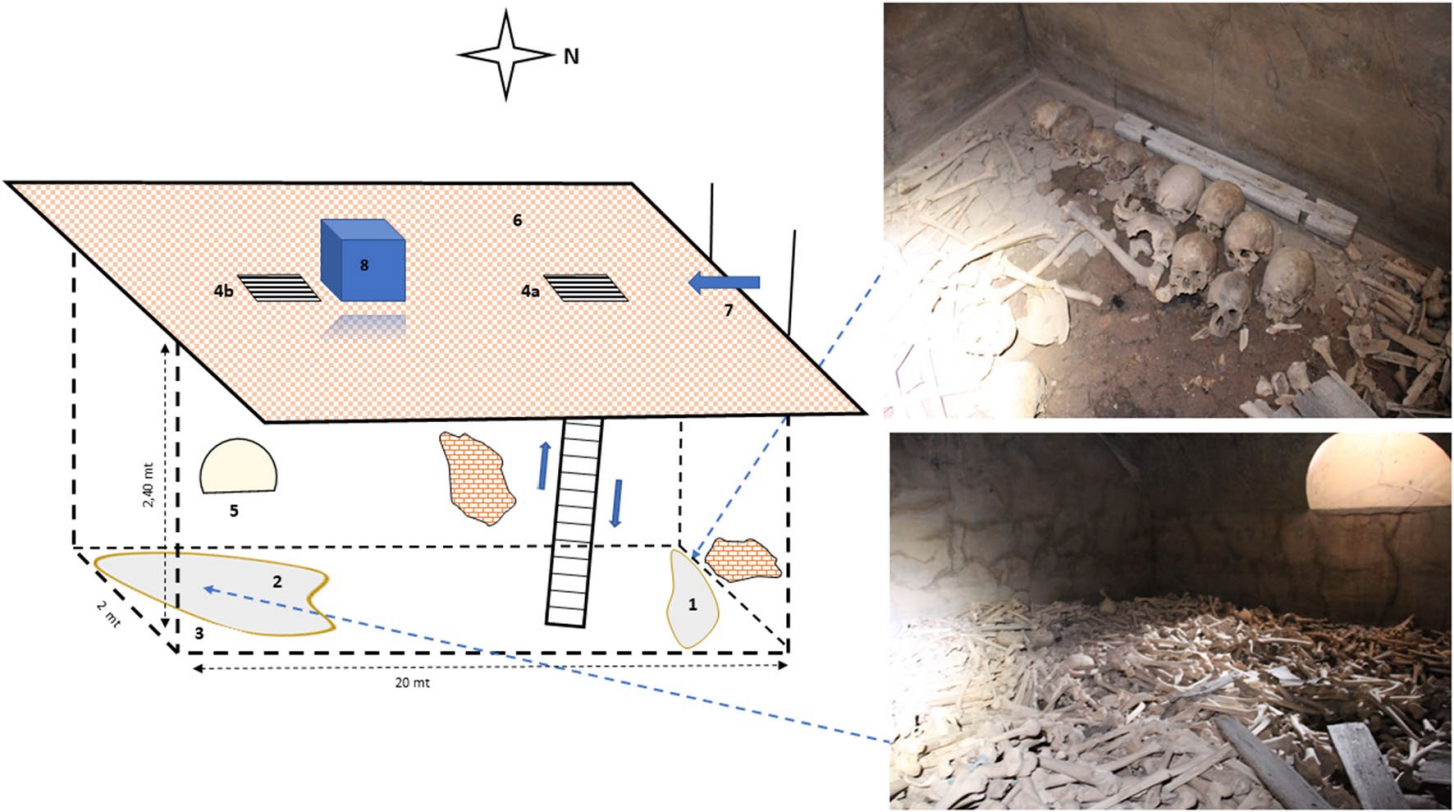
Axonometric projection of the charnel house of the Villaputzu site. Schematic representation of the charnel house used as a sampling site of historical dental *calcoli*: 1 pile of skeletal material in the recovery stratum; 2 walking surface; 3 buried muddy stratum; 4a NW trapdoor; 4b SE trapdoor; 5 air ducts; 6 church floor; 7 church gate; 8 altar church; x = sample recovery spot. Drawn by G. Orru’.

The charnel house (Fig. 1) is an underground chamber located under the church in Villaputzu’s cemetery (39° 26′ 13. 7″ N–9° 34′ 08. 3″ E; URL maps in supporting material). The whole complex was built in the nineteenth century and remained usable until 1901. After human remains were exhumed from their original graves, they were transferred and thrown into the charnel house through the NW trapdoor. This caused the bones to be scattered around the room, and many of them showed fall-related lesions. To conduct the recovery operations of the human material, the personnel in charge wore appropriate protective attire consisting of coveralls, gloves and dust masks. Following the research purpose, operators focused on the salvage of preserved *crania* and *mandibulae*. The supragingival and subgingival *calculus* was collected and combined from the teeth of 12 adult human remains, whose identity was confirmed by morphological observation by an archeologist. Both photographic and written records were made in situ*,* and a code was provided for each sample. Samples were then stored in sterile containers to avoid contamination and transferred to the laboratory for biomolecular analyses. Periodontal bacterial DNA (pbDNA) was strictly confined inside a strong mineralized coat, which made pbDNA cross-contamination between skeletal remains highly improbable in the field. However, all necessary precautions were taken to avoid any possible cross DNA contamination. The samples (*mandibulae* and *crania*) were gently collected to avoid stirring up the dust inside the charnel house. In addition, a small amount of the soil around the skeletal remains was collected in sterile Falcon™ tubes (BD Biosciences Bedford MA-USA) and used as an internal negative control to ensure effective control of the contamination level and avoid experimental artefacts.

Morphological examination of the teeth and the first step of DNA extraction and the preparation of the qPCR master mix were carried out under a laminar flow cabinet by operators wearing sterile gloves and surgical masks. DNA AWAY™ Surface Decontaminant (Thermo Fisher Scientific) and UVA light were used to degrade any possible source of unwanted DNA deposited on the disposables and work surface.

### Dental samples

**Figure 2:**
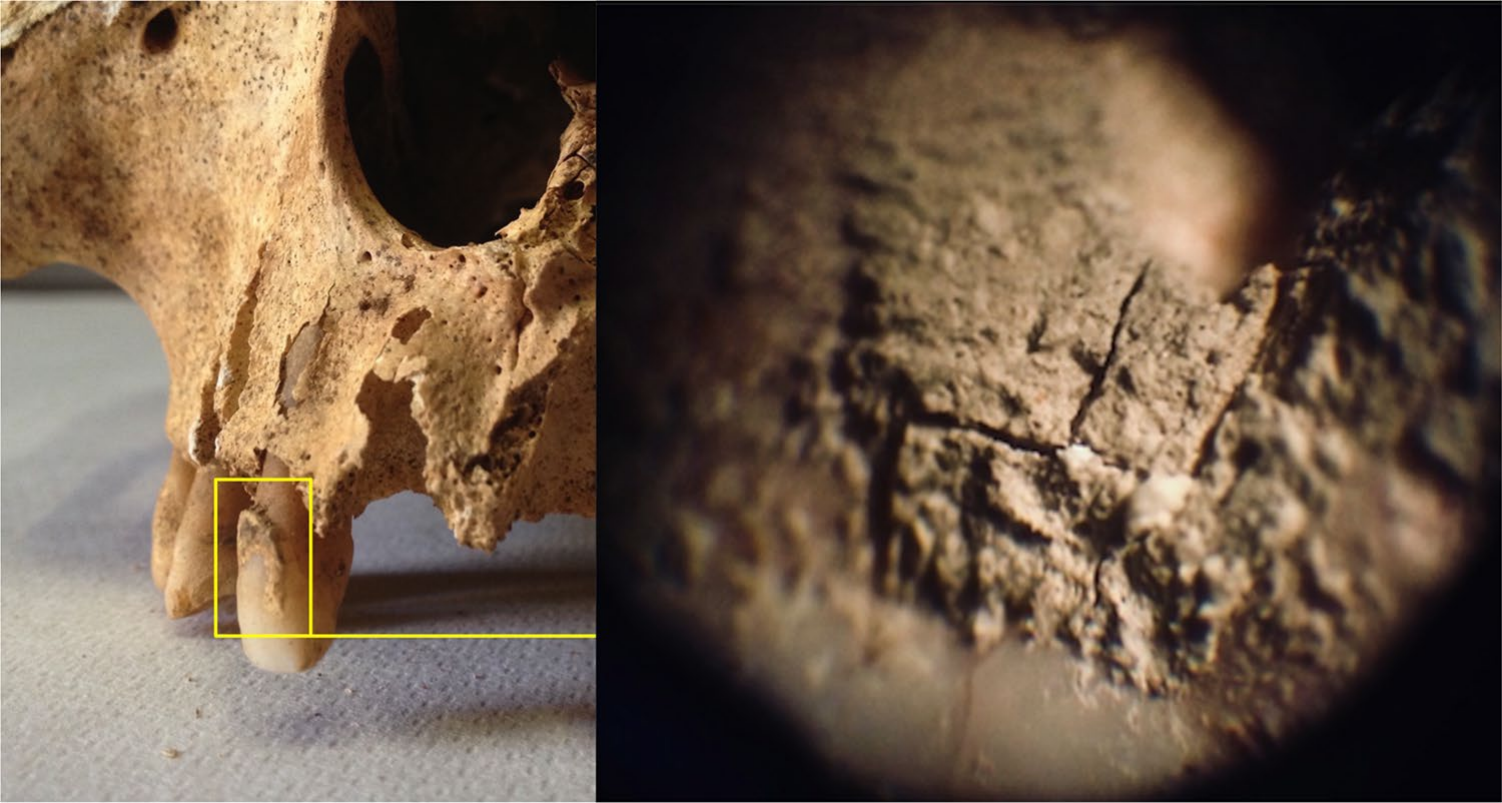
Dental *calculus*. Dental *calculus* in an ancient masculine maxillary left lateral incisor and its structure magnified under a light microscope (50×).

Two different sample groups (Table S1) were analysed, including contemporary living individuals (modern group n = 26) and historical individuals (historic group n = 12). The modern group included the analysis of a set of 26 recent teeth samples from 12 male and 14 female subjects aged between 18 and 75 (mean 48.9 and median 51.0) recruited from the University of Cagliari, Department of Dental Disease Prevention (DDP), during routine dental extraction procedures. Individuals belonging to the modern group did not present microbial dysbiosis or evident clinical signs of periodontal disease. These specimens were chosen from teeth that were non restorable due to trauma or during full mouth reconstruction. These samples were extracted before this study and date back 2–10 years. Before any tooth extraction, patients were routinely asked to sign an informed consent for further use of the extracted teeth for research purposes. Sex and age were registered after dental extraction for each sample. Each tooth was placed and stored in a sterile ø 60 mm polystyrene Petri Dish (Nunch, Thermo Fisher Scientific Inc. Waltham, MA, USA) at room temperature and kept in dark and dry conditions until analyzed. The historical group included 12 individuals (9 *mandibulae* and 3 *crania*), 7 females and 5 males aged between 18 and 50 years. A total of 51 teeth were extracted from 12 adult human skeletons, with a maximum of none teeth and a minimum of one per individual, as described for each individual in Table S1. Assuming the teeth coming from the same oral cavity were related, supragingival and subgingival *calculi* coming from the same historic individual were collected and combined to increase the probability of analytical success as described by Shiba et al.^23^. The samples were gently cleaned with a cotton swab to remove surface dirt. The presence of periodontal diseases or caries were recorded for each *cranium*. Only the teeth containing visible *calculus* (Fig. 2) were considered for biomolecular analysis, and the mean was two visible *calculi* per tooth. The teeth were then extracted from the dental alveoli of the respective mandibles by using sterile equipment.

Teeth surfaces analysis was performed with a binocular microscope, with a magnification of 10×–50×^25^, to assess the presence of *calculus*, wear, hypoplasia and caries lesions, or horizontal bone loss by direct visual measurement (DVM) in *mandibulae*^26^.

### Teeth pretreatment

Teeth pretreatment was performed to prepare the historical and modern teeth for DNA extraction. After visual inspection, all samples underwent a modified cleaning-decontamination procedure based on Bolnick et al.’s method^27^. Briefly, teeth were first brushed with single-use toothbrushes in 2 mL nuclease-free H_2_O (Invitrogen) to remove soil or traces of dental plaque, and then dipped in 2 mL 5% w/v sodium hypochlorite (NaOCl) for 20 min (Masnata Chimici, Elmas-Cagliari Italy). Samples were exposed to a UV lamp at 254 nm for 10 min on each side to eliminate any possible surface DNA contamination. The teeth were then transferred into 15 mL Falcon™ tubes (BD Biosciences Bedford MA-USA) containing silica spheres Ø = 2 mm to allow dental *calculus* mechanical disruption/breakdown. A solution containing EDTA 0.5 M pH 8.0 (1 mL), and 0.025 mL of 10 mg/mL proteinase K solution (Sigma Aldrich) was added to each tube. Afterwards, the samples were incubated overnight at room temperature in a rotary suspension mixer (New Brunswick Scientific, Connecticut USA, model tc-6) set at 56 rpm with a 45° angle of inclination (Fig. S1). The following day, the dental *calculus* suspension from each sample was used for DNA extraction.

### DNA extraction from dental *calculus*

Bacterial DNA from the *calculus* suspensions was extracted using the CTAB method^28^. Briefly, 0.4 mL of suspension was collected in 1.5 mL tubes and incubated for 30 min at 85 °C. Then, 0.05 mL of 1 mg/ml lysozyme (SIGMA—Aldrich, ST. Louis, Missouri, USA) were added, and the samples were incubated at 37 °C for 1 h, at − 20 °C for 30 min and finally at 65 °C for 10 min. Then, 0.07 mL of 10% sodium dodecyl sulfate (SDS) and 0.005 mL of proteinase K at a 10 mg/mL concentration (SIGMA—Aldrich) were added. After vigorous vortexing, the mixture was incubated for 10 min at 65 °C. Next, 0.1 mL of NaCl [5 M] and 0.1 mL of CTAB/NaCl (0.274 M CTAB, hexadecyl trimethylammonium bromide and 0.877 M NaCl, Sigma-Aldrich) were added to the tube, which was gently mixed by inverting and then incubated at 65 °C for 10 min. Then, 0.75 mL of SEVAG Chloroform: Isoamyl Alcohol 24:1 (Sigma-Aldrich) was added, and the mixture was vortexed for 10 s. After centrifuging for 5 min (at 12,000 RCF), the upper liquid phase containing DNA was transferred to an ice-cold tube. Then, 0.45 mL of iced isopropanol (Sigma-Aldrich) chilled to − 20 °C was added to the supernatant, and after mixing gently, the samples were incubated for at least 1 h at − 20 °C and centrifuged at 12,000 RCF for 30 min at 4 °C. Then, the supernatant was carefully removed. Tubes were kept open for approximately 10 min to allow isopropanol evaporation. DNA pellet was suspended in 20 μl of 1X TE Buffer (Thermo Fisher Scientific Inc. USA). DNA concentration and purity (260/280 and 260/230 nm ratio) were measured by a NanoDrop™ spectrophotometer (Thermo Fisher Scientific Inc. USA). DNA (0.5 ng) from each extract was used for RT-PCR. To remove the residual calcium salts, samples containing DNA were treated with a further purification step with a Wizard®SV Gel and PCR Clean-Up System kit (Promega). The DNA was eluted in 0.05 mL of ultrapure DNase/RNase-free distilled water (Gibco, Invitrogen Paisley, Scotland, UK). The samples were stored at − 80 °C before use, and 0.002 mL was used as a DNA suspension for RT-PCR.

### Oligonucleotide design for RT-PCR

RT-PCR primers species-specific for the detection of PB were designed by a blasting search in GenBank (https://blast.ncbi.nlm.nih.gov/Blast.cgi). Oligonucleotide primers and accession numbers for corresponding sequences and thermodynamic values are shown in Tables S2 and S3 respectively. PCR oligos design included the verification of RT-PCR efficiency, annealing temperature (Ta) (UNAFold was calculated with DINAMelt Server^29^), primer secondary structure (dimer formation, self-complementarity and hairpin structures) with low free energy (− ΔG)^29^ oligo were calculated using the Oligo program version 4 (MedProbe, Oslo, Norway) and Mfold program^30^ to have the most similar Ta and thermodynamic properties for all oligos pairs. Primer specificity was checked in silico by blasting the primer sequences to related species (data not shown). To quantify the total bacterial load^31^ present in dental *calculus*, 16S rRNA primers were designed to target the conserved regions C3 and C4 of the 16S rRNA gene^31,32^ as, represented in Fig. S2.

### Detection and quantification of PB

#### Positive controls

DNA was extracted from different PB strains, including *Aggregatibacter actinomycetemcomitans*, genotype 652, CCUG 37005 (68) (Culture Collection, University of Göteborg, Sweden), *Fusobacterium nucleatum, subsp. nucleatum CCUG 32989, Prevotella intermedia* CCUG 2404, and *Porphyromonas gingivalis* CCUG 25893. *Peptostreptococcus micros CCUG 46357*, *Treponema denticola* DSMZ 14222-Deutsche *Sammlung von Mikroorganismen*, Braunschweig, Germany, *Tannerella forsythia* cip 105220 (Institute Pasteur, Paris, France). DNA extracted from *Escherichia coli* ATCC 7075 (American Type Culture Collection) was used as a reference for the quantification of total bacterial load by RT-PCR since the extraction of DNA from *E. coli* guaranteed a high yield and quality of DNA to perform standard curves.

All strains (except *E. coli*) were cultured on CDC Anaerobe 5% Sheep Blood Agar plates (Microbiol, Uta, Cagliari, Italy), and incubated in an anaerobic jar for 7 days at 37 °C. *E. coli* was inoculated onto a Muller Hinton Broth agar plate at 37 °C in air for 24 h. Some isolated colonies of each strain were suspended in distilled water until a McFarland turbidity of 4 units, corresponding to approximately 1.2 × 10^9^ CFU/ml. A total of 400 µl of these suspensions was used for the CTAB DNA extraction procedure, as previously described. Bacterial DNA was thus quantified by using a Qubit fluorometer (Thermo Fisher Scientific-USA). Each RT-PCR standard curve consisted of six concentrations made by tenfold serial dilutions, ranging from 10^7^ to 10^2^ genomes/μl for each bacterial species. A total of 5.1 × 10^7^ fg/µl of DNA from *E. coli* contained 1 × 10^7^ genomes per ul (*E. coli* genome length = 4.7 × 10^7^ bp) (https://www.technologynetworks.com/tn/tools/copynumbercalculator). The detection limit of our qPCR assay ranged from 2 × 10^3^ to 4 × 10^2^ fg/ul of DNA, corresponding to 400 and 100 genome per µl respectively.

#### Negative controls

An environmental control for RT-PCR was included in the analysis and consisted of approx. 5 g of soil collected near the skulls before the sampling. The soil material was included in the bacterial DNA extraction and each RT-PCR analysis along with the other teeth samples. In addition, an RT-PCR negative control containing only PCR mix plus nuclease-free water was included in each RT-PCR run.

#### RT-PCR and standard curve conditions

The concentrations of PB pathogens and the total bacterial load in the samples were calculated using RT-PCR respective to a standard curve obtained for each pathogen. The quantity of each bacterium (expressed as genomes/µl) was calculated by the interpolation of the sample cycle threshold (CT). Each pathogen was expressed as a % of DNA suspension by using the following formula:

$$\% \text{Pathogen}= \left(\frac{\left[\text{N}^\circ \text{ Pathogen genomes}/\mu \text{l}\right]}{\left[\text{Total Bacteria genomes}/\mu\text{l}\right]}\right)*100$$

The total bacterial genome titer was obtained by using the bacterial PCR threshold cycle interpolated from the *E. coli* standard curve. RT-PCR was performed with a LightCycler instrument (Roche Diagnostics Mannheim, Germany) and SYBR Premix Ex Taq Kit (TaKaRa-Clontech®) according to the manufacturer's instructions. The 0.02 mL final volume contained 1X 1XPremix Ex Taq (2X), 1X SYBR Green (10,000X), 0.22 μM of each primer, and 1 to 10 ng of DNA extract. The thermoprofile of qPCR consisted of (1) on denaturation step at 95 °C for 30 s, (2) followed by 40 cycles of 5 s at 95 °C, 30 s at 60 °C, and 20 s at 80 °C. The melting curve was performed from 45 to 95 °C and with a transition rates of the 0.1 °C/s in the 45 °C segment and 20 °C/s for the other steps. Fluorescence was detected at the end of the 80 °C segment in the PCR step (single mode) and the 45 °C segment in the melting step (continuous mode) in the F1 channel. During the optimization of the RT-PCR reaction, products were analyzed by using agarose gel and RT-PCR melting curve analysis to ensure a correct sample product size and melting temperature (Tm) of each amplicon (Figs. S3 and S4). Melting curve analysis relies on the principle that dsDNA products of different sizes or compositions melt at different temperatures, producing distinct melt peaks. Melting curve analysis of the RT-PCR amplicons for each PB showed that intercalating dye (SYBR Green) produced a single, specific product (presence of a single peak above at Tm of 80 °C) (Figs. S3 and S4). All RT-PCRs, except for sex determination, showed a comparable efficiency from ≃ 96 to 98%, Table S3. In addition, each pair of RT-PCR primers was tested using DNA template extracted from each PB species to verify the amplification product only in the presence of the specific DNA target.

RT-PCR efficiency was evaluated by using each standard curve slope with the equation [e = 10^−1/slope^], where e = theoretical efficiency, slope of the standard curve, plotted with the y-axis as Ct and the x-axis as log quantity of bacterial genomes.

To avoid errors due to different sampling or different amounts of *calculus* for each tooth, the total bacterial load (number of corresponding *E. coli* genomes) was normalized per microgram of DNA in the sample, quantified by the Qubit 9 V^®^ fluorometer (Invitrogen).

### Sex determination

Regarding the archaeological samples, sex determination was carried out by the RT-PCR procedure described by Fazi et al.^33^, using primers/probe described in Table S2, as well as in Figs. S5 and S6. Mandibular landmarks were also used as sexual dimorphism indicators^34^. The concordance between the two sex determination methods was 99%. Concerning modern samples, sex was recorded alongside the extraction procedure.

### Age estimation

To estimate the age of the historical teeth two methods were applied: (1) one based on the use of a dental eruption chart, and (2) the second method named, Cameriere’s method based on morphological variations of teeth^5^. Briefly, Cameriere’s method consists in the measurement of the ratio between the length of the projection of the open apices in the tooth roots/the length of the axis major of the tooth. The age measurement was based on the ratio between the length of the projection of the open apices and the length of the axis major of the tooth area/pulp area^36^.

### Statistical analysis

Absolute quantitation by RT-PCR was performed by procedures described in the literature^37^. Each sample was analyzed in three separate RT-PCR runs in duplicate and quantitative data were expressed as the mean ± SD. Pearson's chi-squared and Mann–Whitney U tests were used to evaluate the significance between different analytical groups. modern/old or males/females (*p < 0.05, **< *p* 0.01, ***p < 0.001). The accordance level between anthropological and biomolecular methods (sex determination) was estimated by the Cohen statistical procedure^36^.

## Results

### Dental caries and total bacterial load among historical and modern dental samples

**Figure 3:**
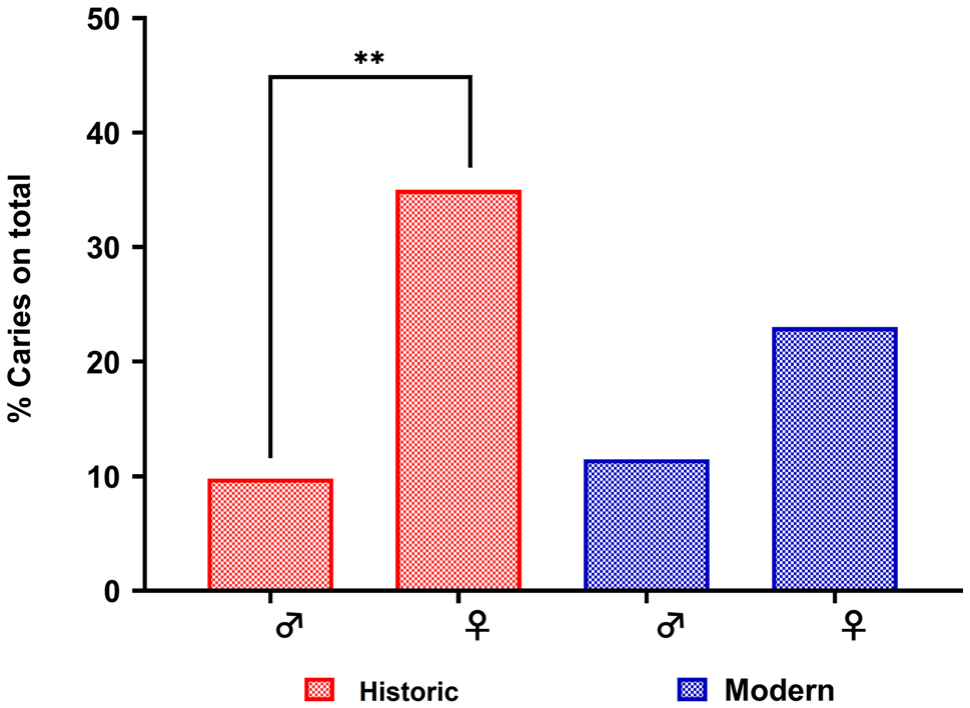
Sex differences in dental caries % evaluated in ancient and modern teeth. Total historical teeth n = 51: historical male teeth n = 23; historical female teeth n = 28; total modern teeth n = 26: modern male teeth n = 12; modern female teeth n = 14 (*p* < 0.05).

Macroscopic investigation of dental *calculus* revealed the presence of 45.1% carious lesions in historical teeth (number of decayed teeth/total teeth), whereas modern teeth presented 34.6% dental caries (Table 1, *p* > 0.05). In particular, historical females (teeth n = 28) showed a significant increase in caries (35.3%), compared to the 9.8% in males (teeth n = 23) (*p* < 0.05) (Table 1, Fig. 3). In contrast, no significant difference in the prevalence of caries was observed between sexes in the modern group (*p* > 0.05).

**Table 1:** Macroscopic investigation of dental *calculus* of the examined skeletal fragments, teeth and mandibulae (% per gender) coming from historical and modern individuals.

Lesion	Total individuals	Males	Females	Total individuals	Males	Females
% dento-alveolar features in historical teeth				% dento-alveolar features teeth in Modern teeth		
Caries*	45.13	9.84	35.35	34.62	25.00	42.86
Tooth Wear	50.98	39.13	60.71	38.46	33.33	42.86
Hypoplasia	35.29	11.35	14.57	0	0	0
Bone loss^§^	33.33	40.00	28.57	nd	nd	nd

The macroscopic investigation of dental *calculus* was performed per tooth and describes the frequencies of dento-alveolar features by sex.

Total historical teeth n = 51; Historical males teeth n = 23; Historical females teeth n = 28.

Total modern teeth n = 26 Modern males teeth n = 12; Modern females teeth n = 14.

*% carious lesions in teeth (number of decayed teeth/total teeth).

^§^Data related to alveolar reabsorption.

Morphological examinations highlighted a poor oral health status with a higher incidence of bone loss (33.3% of individuals) and tooth wear (51% of teeth) in historical samples (Table 1). Furthermore, the presence of enamel hypoplasia (35.2% of individuals) could reflect exposure to systemic stress events (Table 1). Representative images obtained with ancient skeletal remains (teeth and mandibulae) are shown in the supporting figures: Figs. S7–S11.

**Figure 4:**
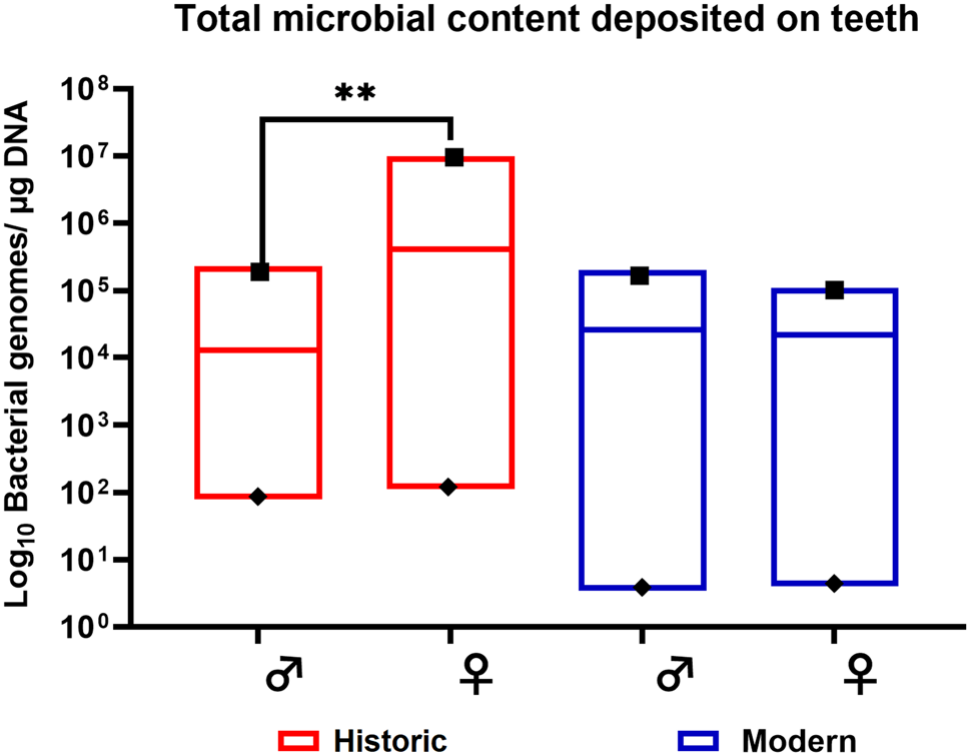
Amount of total bacterial genomes per µg DNA. The historical samples belonging to the females’ teeth group showed a remarkable total bacterial variation in max–min and median values in comparison with all other groups (Non-parametric test U di Mann–Whitney test). Historical males n = 5, historical females n = 7; modern males n = 12, and modern females n = 14) (*p* < 0.05).

Total DNA was successfully extracted from the mineralized concretions of dental *calculus* and teeth of historical and modern individuals and used to determine the total microbial load by 16S rRNA gene quantification in RT-PCR^31^. While no variation in total bacterial load (bacterial genomes/µg DNA) was observed between males and females in modern individuals (*p* > 0.05), a difference was measured between sexes among ancient samples (historical males n = 5, historical females n = 7, modern males n = 12, and modern females n = 14). As shown in Fig. 4, the total bacterial load in the ancient females’ teeth varied between 1.13 × 10^2^ and 1.0 × 10^7^ (with an average of 4.1 × 10^5^), while in ancient males’ teeth ranged from 7.8 × 10^1^ to 2.3 × 10^5^ (with an average of 1.30 × 10^4^). In contrast, no significant difference was observed among the sexes of the modern group (*p* > 0.05).

### PbDNA prevalence in historical and modern teeth

Seven PB indicators of periodontal diseases were searched by RT-PCR in dental samples to assess the oral health status of historical and modern individuals.

**Figure 5:**
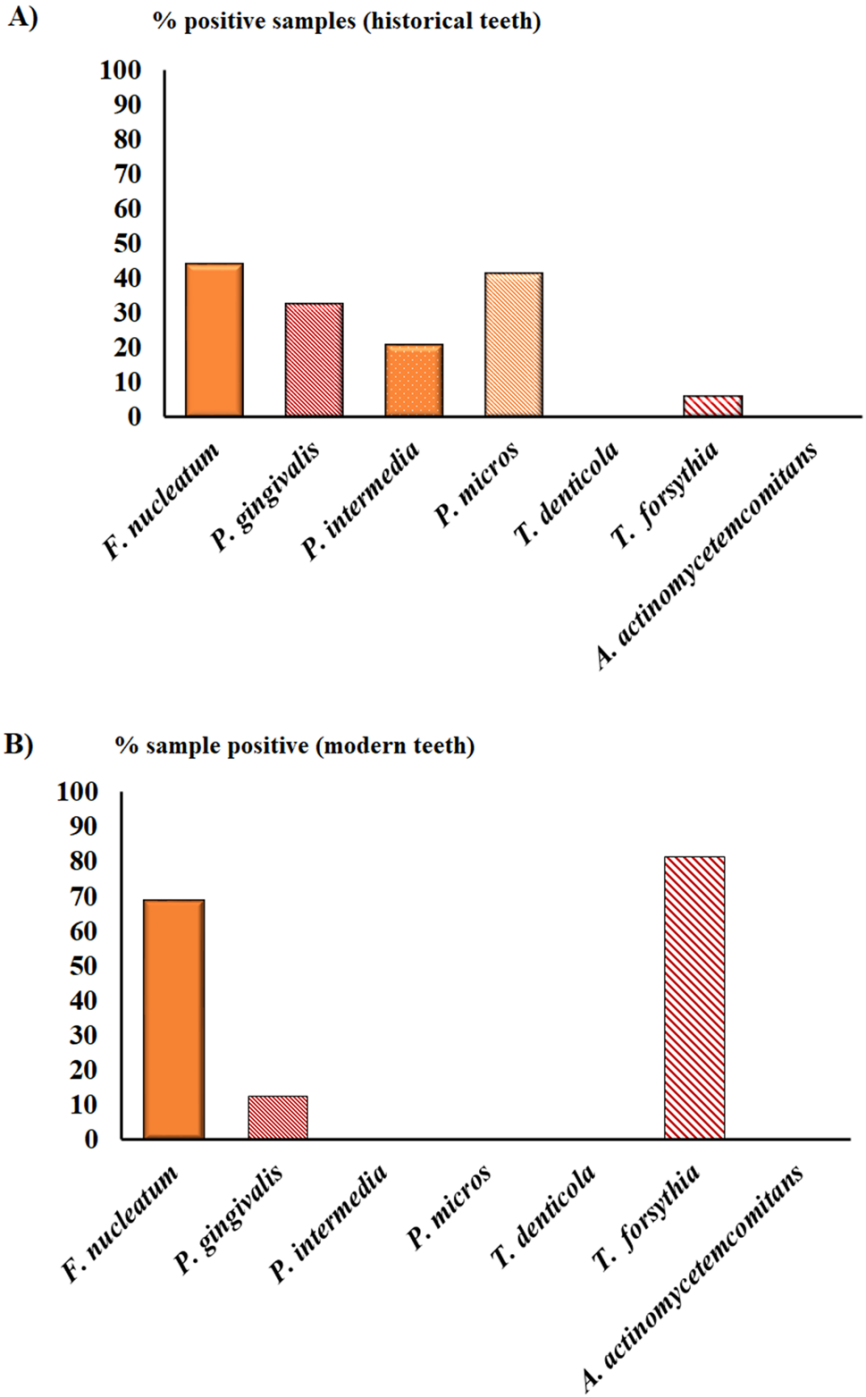
Real-time PCR results. Percentage positive of seven representative periodontal pathogens tested in historical (**A**) and modern groups (**B**). Historical males n = 5, historical females n = 7; modern males n = 12, and modern females n = 14.

As shown in Fig. 5A,B, the presence of each PB bacterium varied between historical and modern groups. *F. nucleatum* and *P. micros* were the most frequently detected bacteria in historical teeth, with 44.1% and 41.1% positive samples, respectively. While bacterial species belonging to Socransky’s “*Red complex bacteria*” were detected in historical teeth, however their presence was not greater than 32.3%, as reported for *P. gingivalis*.

In contrast, *F. nucleatum* was often detected in modern teeth, resulting in 68.7% of the positive samples. However, *T. forsythia* had the highest prevalence, measuring 81.2% of the positive samples in the modern group. This bacterial species representing *Red complex bacteria* was reported to have the most substantial increase in prevalence from ancient to modern individuals. No significant differences were observed for *F. nucleatum* and *T. forsythia* between the male and female subgroups (*p* > 0.05). *A. actinomycetemcomitans* was not detected in any dental specimens (Fig. 5A,B).

### Quantification of PbDNA in dental *calculus*

A remarkable difference in the amount of PB related to the total oral bacteria (expressed as periodontal bacterium genome % of total bacterial genomes) was found between historical and modern teeth. Indeed, the amount of *T. forsythia* was notably higher in modern teeth with ~ 600-fold increase in genome/copies compared to ancient teeth (*T. forsythia* = 31.7% in modern teeth *vs T. forsythia* = 0.05% in ancient teeth with a *p* < 0.05). Furthermore, another significant difference was reported for *F. nucleatum*, with a 50-fold increase in genome/copies in modern teeth (*F. nucleatum* = 2.06% in modern teeth *vs F. nucleatum* = 0.04% in ancient teeth).

**Figure 6:**
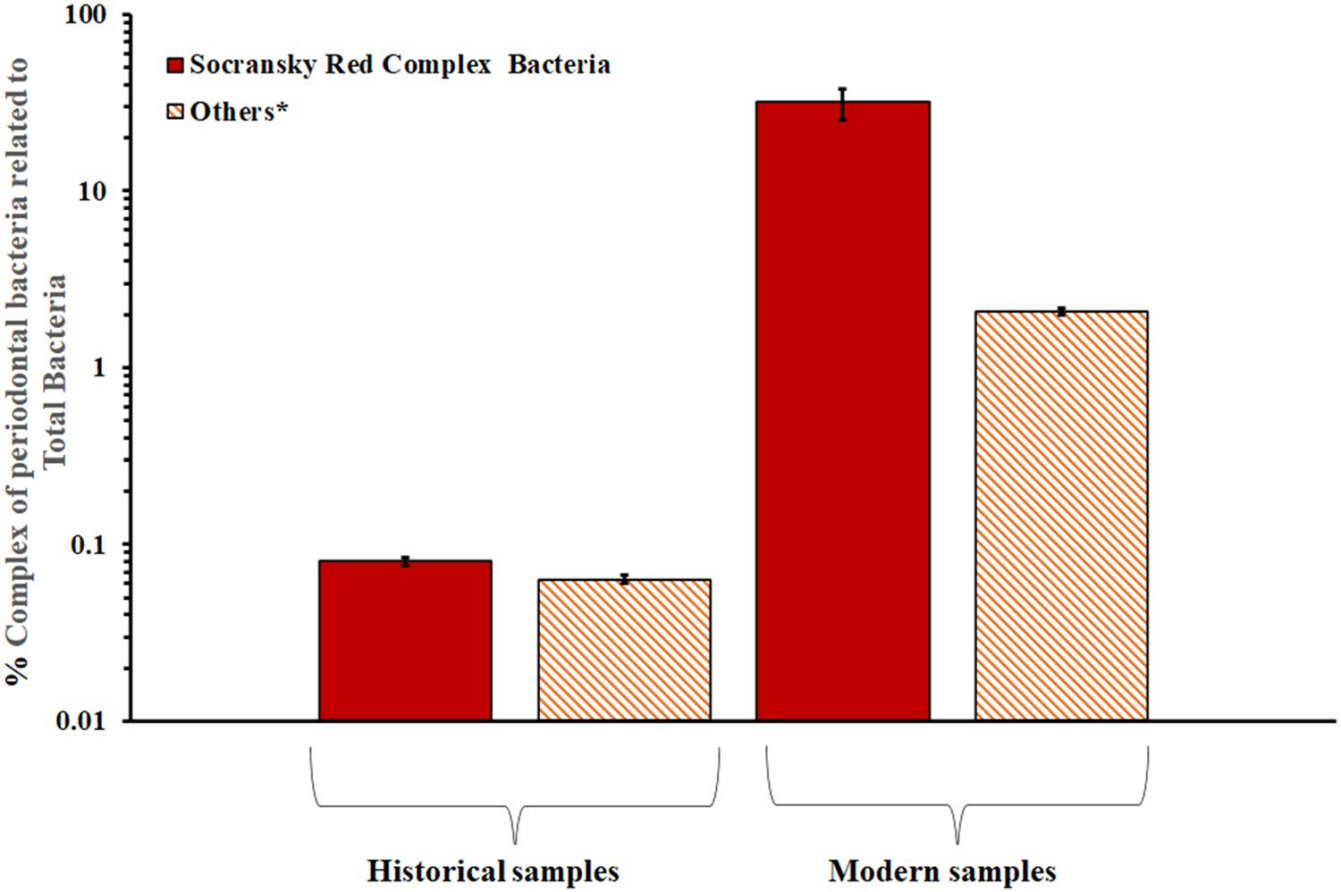
Percentage (%) of periodontal bacterial complexes (Socransky’s Red and partial orange complexes) in relation to the total bacterial load in historical and modern dental samples. The percentage was calculated as the sum of the median concentration values for all teeth for each positive subject. The bars represent the standard error. Historical males n = 5, historical females n = 7, modern males n = 12, and modern females n = 14. Red complex bacteria: % of the quantity of *P. gingivalis*, *T. denticola*, and *T. forsythia* in relation to the total load of bacteria. *Partial Orange complex: % of the quantity of *F. nucleatum, P. intermedia, and P. micros* in relation to the total load of bacteria.

These bacteria were also responsible for the high prevalence of *Red* and *Orange complexes bacteria* in relation to the total bacterial load in the modern individuals (Fig. 6).

No significant difference was observed for PB titer between males and females within either groups (*p* < 0.05, data not shown).

## Discussion

Our study conducted on historical dental *calculus* was based on two assumptions: (1) the macroscopic examination of dental lesions (e.g., caries) represents a valid tool for predicting oral health and human behavior in populations living in different historical periods; (2) the use of modern biomolecular approaches could reveal oral bacterial indicators related to the host’s oral health disease status^38^. Indeed, the study of dental *calculus* of ancient humans is becoming increasingly relevant in archaeological microbiology to delineate both a biological and cultural picture of ancient populations.

To this end, we studied the health status and PB pathogen profile of individuals from a pre-industrial rural community of the early 1800s and modern era who lived in the same region of Sardinian Island. Due to isolated geographical features, we hypothesized that the historical and modern individuals forming the two groups were characterized by low genetic diversity and that differences in oral status were due to environmental and socioeconomic changes over the last 200 years^39^.

The initial morphological analysis of dental lesions already showed an interesting discrepancy in caries frequency between the sexes of both groups. While there were no differences in the frequency of caries between males/females in modern individuals, historical females showed a higher caries frequency than historical males and modern individuals (both males and females) (Fig. 3). Sex-related differences concerning oral health have been extensively described in populations of various geographical origins. Studies conducted in modern developing countries also showed a significant sex discrepancy in dental caries frequency, especially in mature adults, supporting our results on historical Sardinian samples^12,40^. The aetiology of caries in ancient women is complex and could be attributed to multifactorial effects such as changes in sex hormones, dietary habits, culture and gender-based division of labour. These conditions, for example, are more evident in individuals living in developing countries than in developed countries, where differences in oral health conditions between the sexes are usually not reported^41,42^. Similar results were obtained by measuring the bacterial load that turned out higher in historical females compared to historical males of the same era and individuals of the modern era (Fig. 4). These data suggest that the historical females may have been exposed to different living conditions (hygienic and dietary habits) than historical males. Indeed, diet pattern is a pivotal factor that influences the abundance of PB in the oral microbiota^43^. According to historical data^44^, Sardinian communities of the early nineteenth century were sustained by agropastoral economies in which males and females often embraced different roles, lifestyles and dietary habits. Females were mainly involved in the practice of baking with consequent consumption of a diet rich in carbohydrates; this may have caused an increase in dental plaque facilitating the development of caries. In contrast, males used to spend long periods in the countryside subsisting on a protein-rich diet based on meat derived from their livestock.

PBs are located in different niches of the mouth represented by (1) the supragingival region (supragingival plate) and (2) the subgingival region. While the supragingival plaque may be present in normal physiological conditions, the subgingival biofilm is often associated with a severe infectious effect, such as periodontal disease. Detection of PB by RT-PCR showed a large difference between modern and historic groups (Figs. 5A,B and 6); while no substantial variation in PB species was found between the sexes of each group. In particular, *F. nucleatum* and *T. forsythia* had the greatest significant prevalence in modern individuals.

*T. forsythia* is considered the main contributor to the development of periodontitis, as it is the only member of the *Red complex* capable of adhering to the tooth structure above the gingiva^45^ and effectively suppressing the host’s immune response^46^. Although the presence of *T. forsythia* was recorded in dental *calculus* at a low percentage, it was remarkable how *T. forsythia* was detected in 80% of modern individuals, making it the most dominant bacterium among the members of the *Red complex*. Different clinical studies showed that a high prevalence of *T. forsythia* was associated with increased levels of total cholesterol in patients with chronic periodontitis and atherosclerosis^47,48^. This relationship between periodontal inflammation and atherogenesis may suggest that the level of oral *T. forsythia* has increased in the different eras over the last decades due to a change in leading lifestyle leading to a higher risk of "modern diseases", such as cardiovascular diseases. Furthermore, a recent genomic study conducted on ancient PbDNA isolated from dental *calculus* of pre-Hispanic and colonial Mexicans revealed certain genetic variation among *T. forsythia* genomes and acquisition of antibiotic resistance in modern genomes^49^. The acquisition of genetic traits due to the selective pressure of dietary changes and antibiotics usage may also have caused a higher prevalence of more pathogenic *T. forsythia* strains^50^.

Furthermore, as shown in Fig. 6, *Orange* and *Red complex bacteria* increased by 50 and 600 times, respectively, from historical to modern dental samples (Fig. 6). The increase in *Red complex bacteria* could be linked to the role of the microbiome in oral and systemic diseases, such as degenerative autoimmune affections^51^ and gastrointestinal cancer^52^. Although the amount of PB and the prevalence of their respective associated diseases depend on various biological and host habit factors, some of these still lack clarification.

Our results indicate the bacterial species belonging to “Red Complex”, defined as the cause of periodontal disease in the modern times, was more prevalent in the modern individuals. However it is possible that “Red Complex” does not comprise pathogenic bacteria causing periodontitis condition in the historical population. Therefore, the historical population might potentially have had even higher incidence of periodontal disease than the modern one but it was not detected because the historical causative agents are not targeted in our assay.

On the other hand, other authors have recently suggested a potential beneficial activity mediated by some strains of PB bacteria. For instance, Starkenmann et al*.* found that the PB pathogen *F. nucleatum* is involved in the smell-flavor perception of vegetables by the transformation of cysteine-s conjugates into thiols^53^. Aagaard et al. described *F. nucleatum* as a key microorganism in the placental microbiota, and it presents a double aspect in terms of its pathogenicity profile^53^. First, it is associated in preterm birth, but it can also modulate the initial immune response profile in newborns^53^. A deeper genomic analysis of the PB strains might be needed to characterize the real pathogenicity of the oral pathogens found in ancient and modern dental samples.

These preliminary results suggest an increase in indicators of oral PB pathogens as consequent changes towards a modern lifestyle that occurred in the last 100–200 years in which the oral health conditions are influenced by changes in hygiene conditions, lifestyle and dietary habits.

## Conclusions

In recent years, the interaction between archaeology and new procedures in biomolecular biology, such as the study of human microbiomes, has broken new ground in explaining habits and sanitary conditions in ancient populations. By integrating the two research areas, this preliminary study has allowed us to outline both a biological and cultural picture of a population from southeast Sardinia dating back 100–200 years. Undoubtedly, the ongoing research on tooth lesions and the progress of molecular techniques will allow us to obtain more specific information about the human-oral microbe relationship. These studies are also applicable to the biomedical field and may lead to a greater comprehension of the mechanism of the spread and evolution of microbes. In this context, it may be possible to reconstruct the history of plagues and predict the potential development of future illnesses.

## Links

- NCBI Genome: *Escherichia coli*: https://www.ncbi.nlm.nih.gov/genome/?term=escherichia+coli%5Borgn%5D.
- The UNAFold and DINAMelt Web Server: http://unafold.rna.albany.edu/.
- Google maps location of the Villaputzu’s cemetery: https://www.google.com/maps/place/Ex+Cimitero+comunale/@39.4409774,9.5604656,4253m/data=!3m1!1e3!4m9!1m2!2m1!1sVillaputzu%E2%80%99s+cemetery!3m5!1s0x12e098370b9a99e3:0x21721bfadc4eda75!8m2!3d39.4391819!4d9.5800811!15sChdWaWxsYXB1dHp14oCZcyBjZW1ldGVyeZIBCGNlbWV0ZXJ5.

## Supplementary Information

Supplementary Figures.

Supplementary Table S1.

Supplementary Table S2.

Supplementary Table S3.

## Data Availability

The data that support the findings of this study are available on request from the corresponding author.
