# Supplementary material for: Changes in the oral status and periodontal pathogens in a Sardinian rural community from pre-industrial to modern time
Source: Sci Rep. 2022 Sep 23;12:15895. doi: 10.1038/s41598-022-20193-9 (PMC9508227; doi:10.1038/s41598-022-20193-9)
Supplement: Supplementary file 1 — Supplementary Figures. [file 41598_2022_20193_MOESM1_ESM.docx]

**Supplementary figures**

**
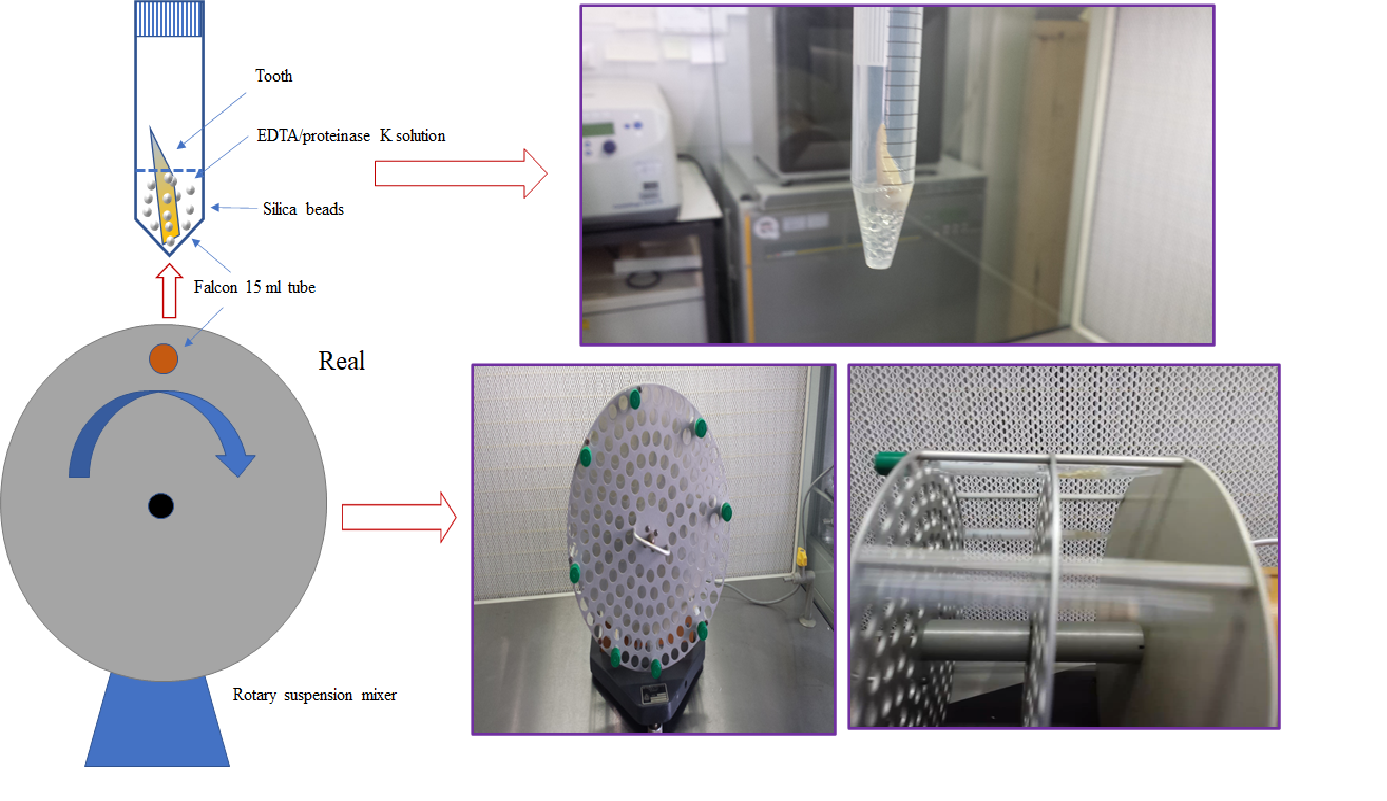
**

**Fig S1.** **Rotary suspension mixer**. Schematic diagram and representative illustrations of the mixer apparatus used for the first teeth pre-treatment to dental calculus mechanical disruption/breakdown. Drawn by G. Orru’.

**
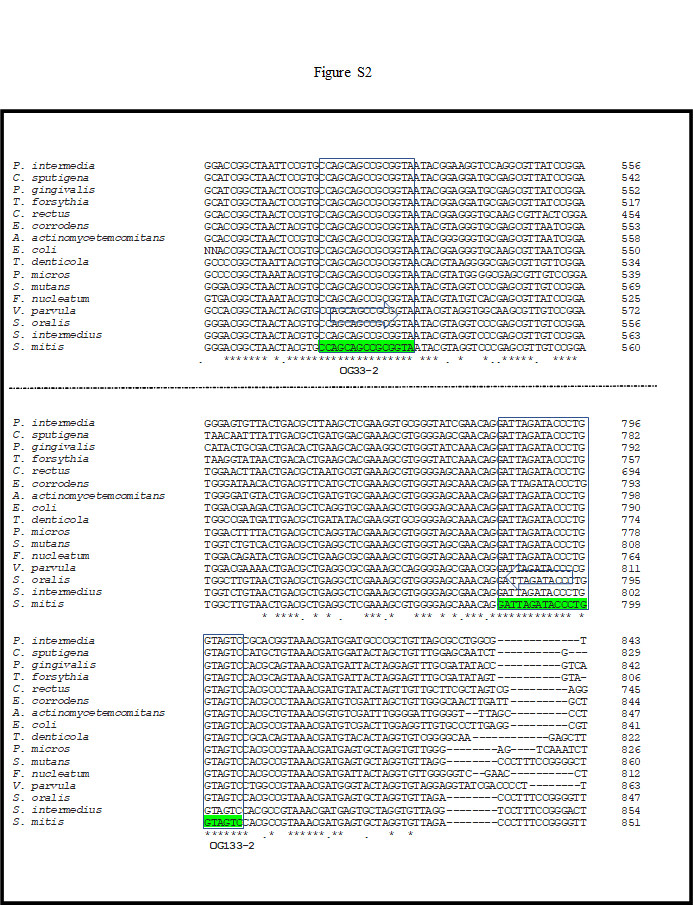
**

**Fig. S2. PCR oligonucleotides used to quantify the total bacterial load.** Common 16S rRNA gene sequence and the PCR primers OG33-2 and OG 133-2 (underlined in green) used to quantify the total bacteria in the dental calculi samples. GenBank accession numbers: M75036, AJ133496, NR_113106, D14143, NR_036899, L16495, X80724, NR_104685, NR_026095, NR_113247, NR_028736, NR_042776, NR_028664.


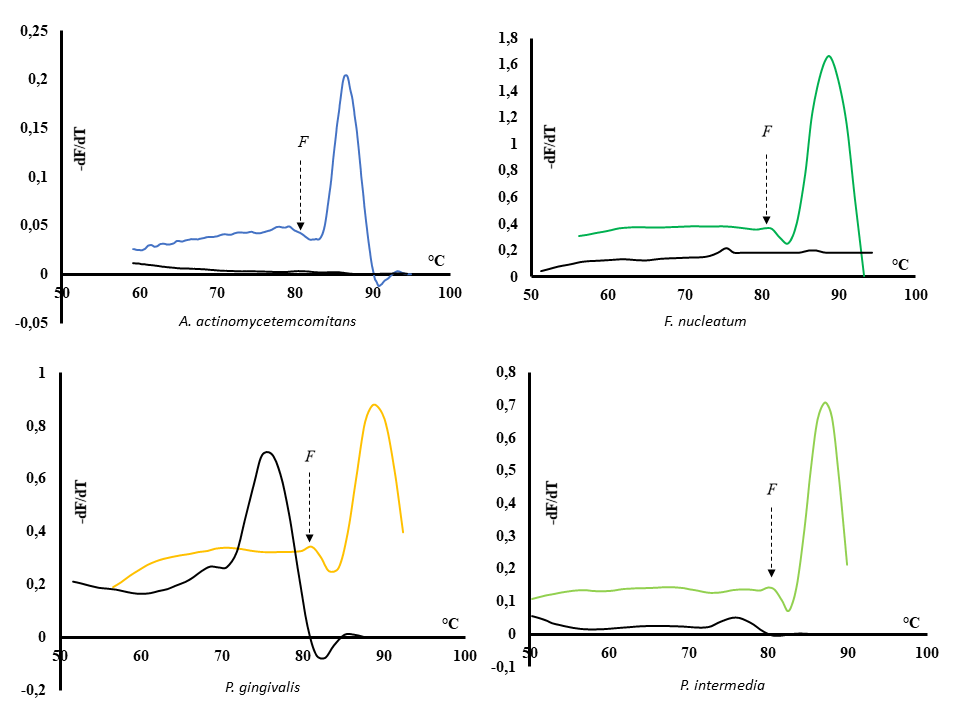


**Fig. S3.** **Real-time PCR melting curves.** Melting peaks obtained by using DNA suspensions from different periodontal *A. actinomycetemcomitans*, *F. nucleatum*, *P. gingivalis*, *P. intermedia*. Black lines represent negative controls, while (F) is the fluorescence reading point (81°C) used in the Real-time PCR protocol.


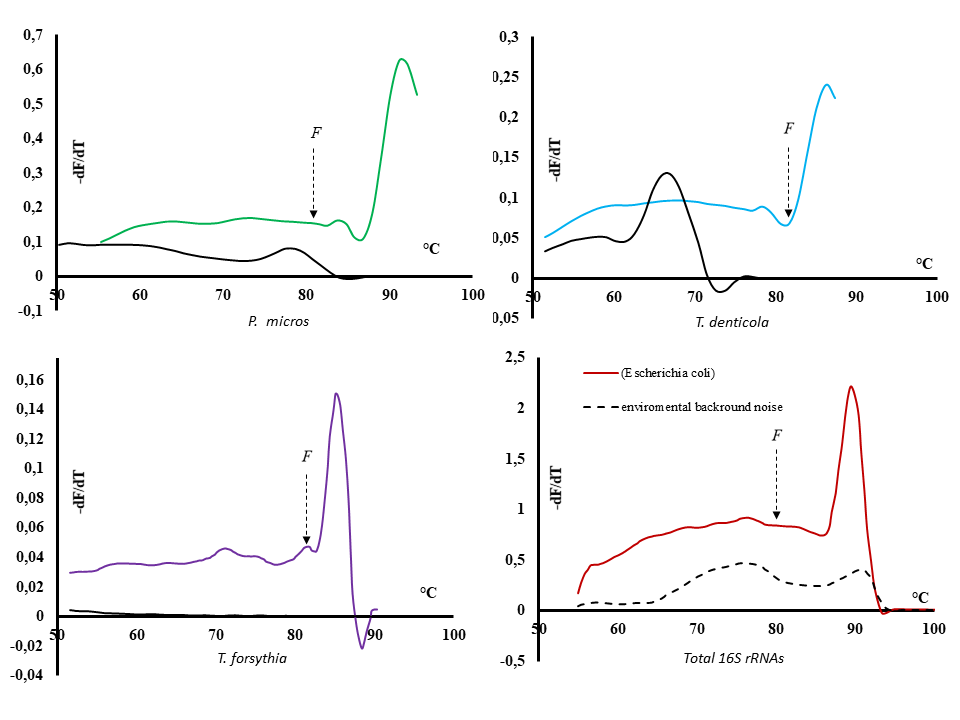


**Fig S4.** **Real-time PCR melting curves.** Melting peaks obtained by using DNA suspensions from *P. micros*, *T. denticola*, *T. forsythia* and total load of 16S rRNA genes (Total bacterial load). Black lines represent negative controls, while (F) is the fluorescence reading point (81°C) used in the Real-time PCR protocol.


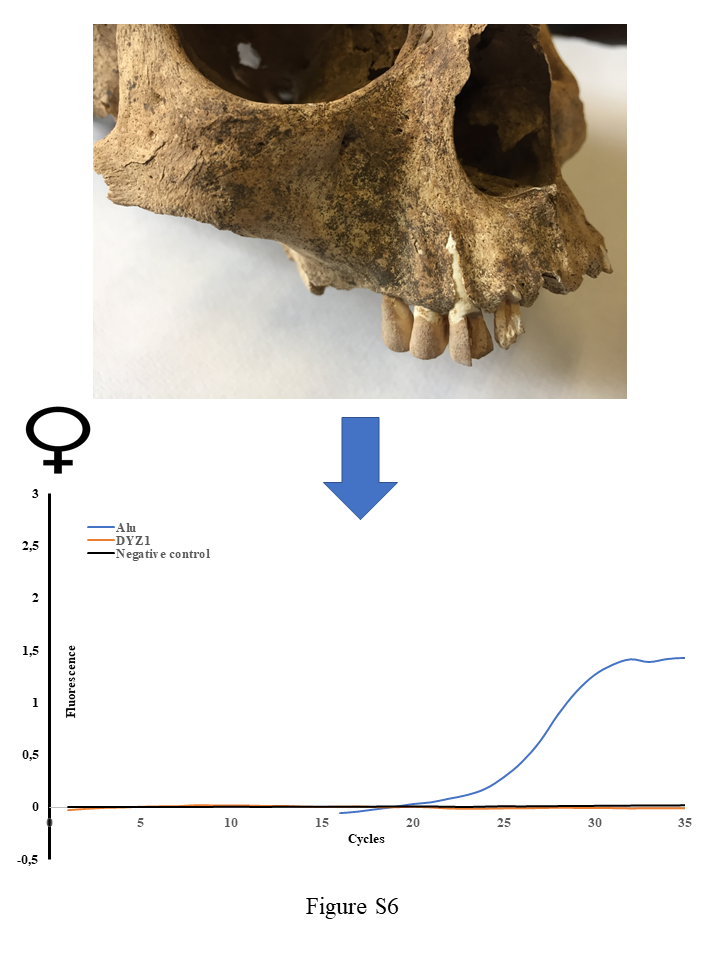

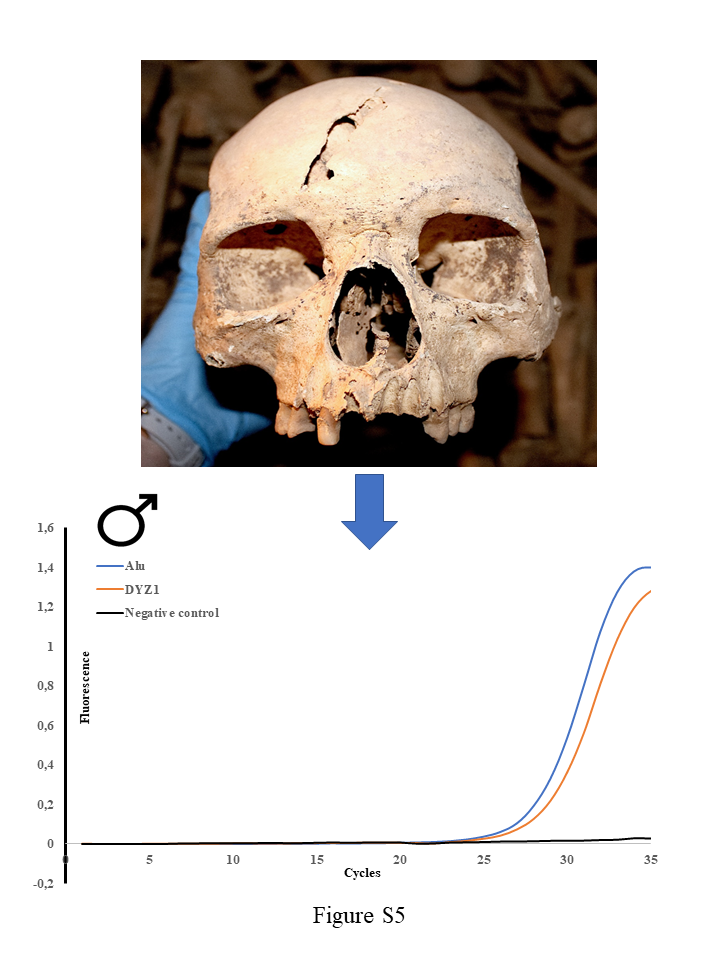


**Figs S5 and Fig. S6. Crania from the charnel house in Villaputzu cemetery**. Fig S5 (left panel): 46 ± 5-year-old male subject (Scarfy) and Fig S6 (right panel) 43 ± 5-year-old female subject (Quasimoda) and their Alu-DYZ1 genetic profile following TaqMan Real-time procedure, described by Fatzi *et al*., (73). The amplification curve was performed by using Light Cycler II instrument (Roche), for this reason, on the contrary to the original protocol, each reaction was performed in separate tubes and TaqMan probes were both labelled with the 6-FAM fluorochrome. Photos taken by G. Orru’

**
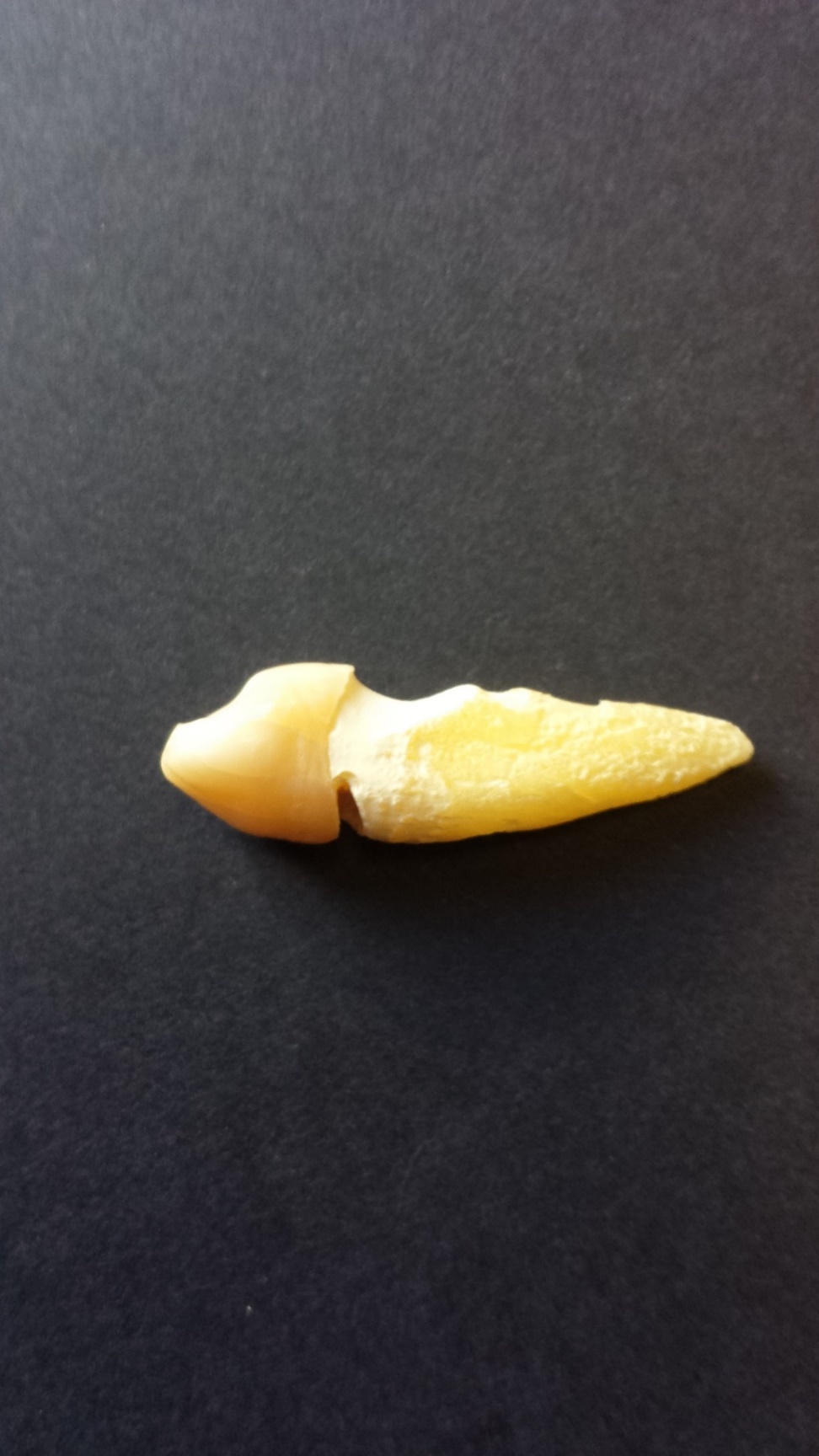

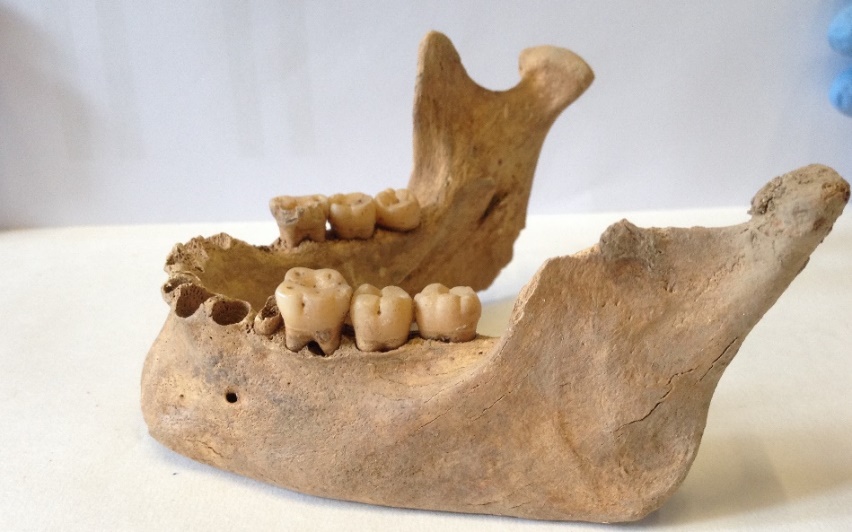
**

**
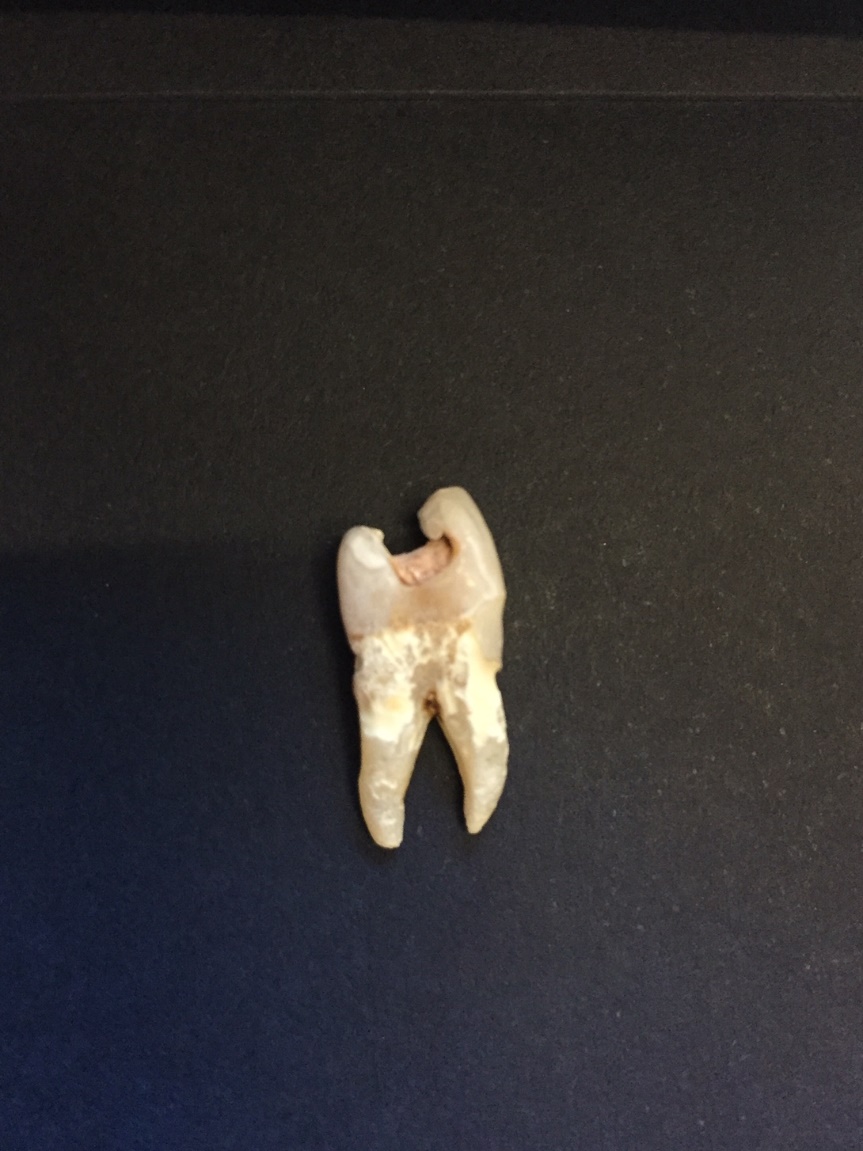

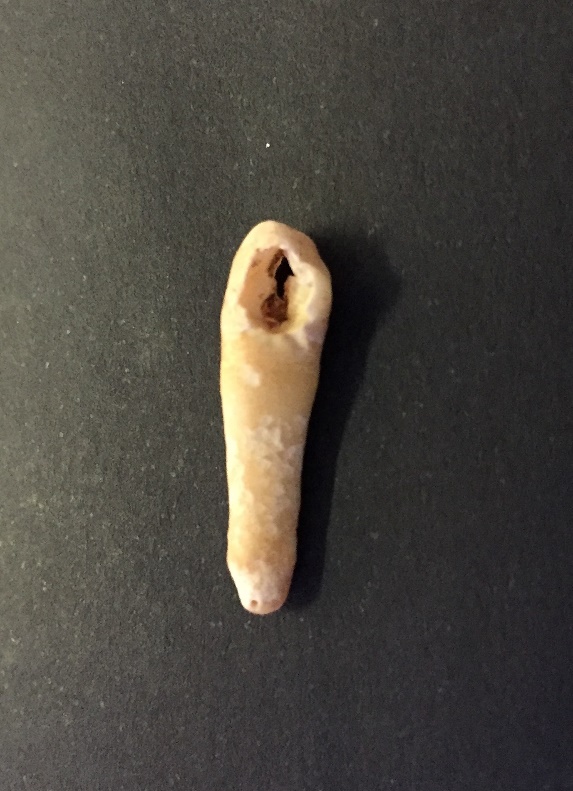
Fig. S7 and Fig. S8. Historical tooth lesions.** Fig. S7 (left panel) shows a close-up view of interproximal dental caries at the level of the cement-enamel junction observed in female subjects. Fig. S8 (right panel) is a photo of the tooth wear of lower molars in an ancient mandibula. Photos taken by G. Orru’

**Fig. S9 and Fig. S10. Historical tooth lesions**. Fig. S9 (left panel) displays severe carious lesions pierced the historical tooth from side to side.

Fig. S10 (right panel) shows a Severe carious lesion in mandibular molar. Photos taken by G. Orru’

**
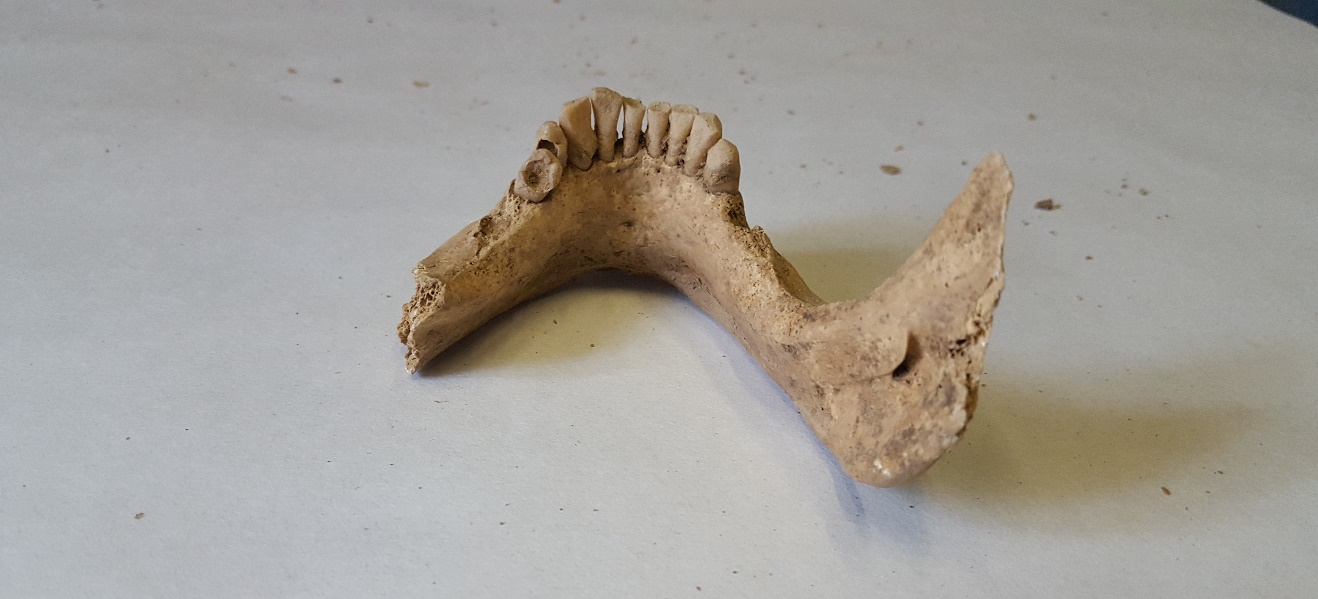
**

**Fig. S11 historical tooth lesions**. Teeth lesions profile in mandibulae from an historical female subject. Photo taken by G. Orru’
