# Supplementary material for: Changes in the oral status and periodontal pathogens in a Sardinian rural community from pre-industrial to modern time
Source: Sci Rep. 2022 Sep 23;12:15895. doi: 10.1038/s41598-022-20193-9 (PMC9508227; doi:10.1038/s41598-022-20193-9)
Supplement: Supplementary file 3 — Supplementary Table S2. [file 41598_2022_20193_MOESM3_ESM.docx]

**Table S2.** Oligonucleotide used in this work for the Real-time PCR.

|  | Oligo sequence 5’------3’ | Oligo name | GenBank accession/gene |
| --- | --- | --- | --- |
| *A. actinomycetemcomitans*^§^ | CCAAGTGTGATTAGGTAGTT | OG43 | M75036/16S rRNA^1^ |
|  | ACCAACCTTCCTCAATAC | OG44 |  |
|  |  |  |  |
| *A. actinomycetemcomitans*^§^ | CATTCTCGGCGAAAAAACTA | OG155 | S68133-M27399 / *LTX* ^1^ |
|  | CCCATAACCAAGCCACATAC | OG156 |  |
|  |  |  |  |
| *F. nucleatum* | GGCCACAAGGGGACTGAGACA | OG41 | AJ133496 / *16 rRNA* |
|  | TTTAGCCGTCACTTCTTCTGTTGG | OG42 |  |
|  |  |  |  |
| *P. intermedia* | CGTATCCAACCTTCCCTCC | OG53 | NR_113106 / 16S rRNA |
|  | ATTAGCCGGTCCTTATTCGAAG | OG54 |  |
|  |  |  |  |
| *P. gingivalis* | GAATCAAATACTTCAGCCGTCT | OG94 | AB006973 / *prtC* |
|  | TTGCAGTTCGTATCGGATCT | OG95 |  |
|  |  |  |  |
| *P. micros* | AAGTGGCGAACGGGTGAG | OG39 | D14143 / *16S rRNA* |
|  | ACAACGCTCGCCCCATAC | OG40 |  |
|  |  |  |  |
| *T. denticola* | AGAGAAAGGGTAATTTGAAG | OG348 | NR_036899/16S rRNA |
|  | TATTATTGTCCCTTCTTTCTT | OG349 |  |
|  |  |  |  |
| *T. forsythia* | GTCGGACTAATACCTCATAAAACA | OG45 | L16495 / *16S rRNA* |
|  | TCGCCCATTGACCAATATT | OG46 |  |
|  |  |  |  |
| Total bacteria count^*^ | CCAGCAGCCGCGGTA | OG33-2 | *16S rRNA* C3 and C4 region^2^ |
|  | GACTACCRGGGTATCTAATC | OG123-2 |  |
|  |  |  |  |
| Sex determination 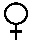 | GAGATCGAGACCATCCCGGCTAAA | Alu (113) F | Alu^3^ |
|  | CTCAGCCTCCCAAGTAGCTG | Alu (113) R |  |
|  | FAM-GGGCGTAGTGGCGGG-DBH1 | Alu probe |  |
|  |  |  |  |
| Sex determination 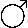 | GGCCTGTCCATTACACTACATTCC | DYZ1 (143) F | DYZ1[^3^](#_ENREF_73) |
|  | GAATTGAATGGAATGGGAACGA | DYZ1 (143) R |  |
|  | 6FAM-ATTCCAATCCATTCCTTT-MGBNFQ | DYZ1 probe |  |

^§^ PCR primer used for *A. actinomycetemcomitans*^1^. Two pairs of primers were used to indentifie two different oral pathogenic genotypes

(652 and JP2)

^*^ Primers 16SrRNA were designed in the conserved regions (C3 and C4) upstream and downstream the variable region V4

**Bibliography**

1. Calabrese, N., Galgut, P. & Mordan, N. Identification of Actinobacillus actinomy-cetemcomitans, Treponema denticola and Porphyromonas gingivalis within human dental calculus: a pilot investigation. J Int Acad Periodontol 9, 118–128 (2007).

2. Ram, J. L., Karim, A. S., Sendler, E. D. & Kato, I. Strategy for microbiome analysis using 16S rRNA gene sequence analysis on the Illumina sequencing platform. Systems Biology in Reproductive Medicine 57, (2011).

3. Fazi, A., Gobeski, B. & Foran, D. Development of two highly sensitive forensic sex determination assays based on human DYZ1 and Alu repetitive DNA elements. Electrophoresis 35, 3028–3035 (2014).
