# Supplementary material for: Changes in the oral status and periodontal pathogens in a Sardinian rural community from pre-industrial to modern time
Source: Sci Rep. 2022 Sep 23;12:15895. doi: 10.1038/s41598-022-20193-9 (PMC9508227; doi:10.1038/s41598-022-20193-9)
Supplement: Supplementary file 4 — Supplementary Table S3. [file 41598_2022_20193_MOESM4_ESM.docx]

**Table S3.** PCR Real-time conditions and thermodynamic parameters

| Detected target | Amplicon length bp | Ta °C * | Amplicon GC % | Efficiency |
| --- | --- | --- | --- | --- |
|  |  |  |  |  |
| *A. actinomycetemcomitans* | 226 | 49/60 | 48 | 96,6 |
|  |  |  |  |  |
| *F. nucleatum* | 182 | 50/60 | 49.5 | 97.2 |
|  |  |  |  |  |
| *P. intermedia* | 389 | 51/60 | 53 | 98 |
|  |  |  |  |  |
| *P. gingivalis* | 143 | 52/60 | 49.7 | 97.1 |
|  |  |  |  |  |
| *P. micros* | 432 | 50/60 | 47 | 97.5 |
|  |  |  |  |  |
| *T. denticola* | 399 | 51/60 | 48 | 96.5 |
|  |  |  |  |  |
| *T. forsythia* | 222 | 52/60 | 51 | 97 |
|  |  |  |  |  |
| Total bacteria (*E. coli*) | 285 | 51/60 | 50.5 | 98 |
|  |  |  |  |  |
| Sex determination 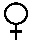 | 113 | 57/60 | 55 | Nd |
|  |  |  |  |  |
| Sex determination 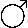 | 143 | 58/60 | 53 | Nd |
|  |  |  |  |  |

The first value represents the theoretical annealing temperature (Ta) calculated by the Oligo^TM^ program, while the second value is the effectively used annealing temperature (Ta) by using Ex Taq (TaKara-Clontech®) Kit recommendations. Nd = not detected.
